# Integrated Multi-Omics Analysis Reveals the Mechanism of Haloxyfop-P-methyl Residue Suppressing Seed Germination in Oilseed Rape (*Brassica napus* L.)

**DOI:** 10.3390/antiox15080988

**Published:** 2026-08-10

**Authors:** Chaochao He, Zhongjing Zhou, Kaige Yi, Yupeng Jiang, Xianling Wang, Zhiqi Ma, Yun Ren, Shuang Liang, Lixi Jiang, Yang Zhu, Shuijin Hua

**Affiliations:** 1 Crop and Nuclear Technology Utilization Research Institute, Zhejiang Academy of Agricultural Sciences, Hangzhou 310021, China; hechaochao@zaas.ac.cn (C.H.); kaige_y0113@163.com (K.Y.); wangxl@zaas.ac.cn (X.W.); mazq@zaas.ac.cn (Z.M.); 2 State Key Laboratory for Quality and Safety of Agro-Products, Zhejiang Academy of Agricultural Sciences, Hangzhou 310021, China; zhouzj@zaas.ac.cn (Z.Z.); liangs@zaas.ac.cn (S.L.); 3 Institute of Crops, Huzhou Academy of Agricultural Sciences, Huzhou 313000, China; yunhuaren@163.com; 4 College of Agriculture and Biotechnology, Zhejiang University, Hangzhou 310058, China; jianglx@mail.zju.edu.cn

**Keywords:** HPM, germination, fatty acid, transcriptome, metabolome, lipidome, rapeseed

## Abstract

Haloxyfop-P-methyl (HPM) is widely used for grass weed control in oilseed rape production. However, whether its residues affect subsequent seed germination and the underlying mechanisms remains unclear. In this study, seeds harvested from HPM-treated fields exhibited a significantly reduced germination rate of 36.5%, compared with 100% in the control group, accompanied by abnormal cell morphology. Residue analysis revealed an HPM concentration of 3.36 mg kg^−1^ in the treated seeds, exceeding the Chinese maximum residue limit (MRL) of 3 mg kg^−1^ (GB 2763). Given that herbicides often induce oxidative stress, we measured a range of antioxidant markers (H_2_O_2_, MDA, SOD, POD, GSH, GSSG, AsA, and DHA) during germination (12 h and 24 h) but detected no significant changes, which did not support oxidative stress as the primary mechanism during this phase. Relative electrical conductivity measurements further indicated that membrane integrity was already compromised in dry seeds, suggesting that oxidative injury—if it occurred—may have taken place during seed development rather than during germination. To elucidate the molecular basis, we performed transcriptomic, metabolomic, and lipidomic analyses, which revealed significant downregulation of fatty acid biosynthesis genes and marked alterations in the lipidome profile. Collectively, our multi-stage investigation demonstrates that HPM residue accumulation is associated with impaired seed germination, primarily through metabolic disruption of lipid reserves rather than oxidative damage during germination, highlighting a non-target phytotoxic effect of this herbicide on the crop itself. These findings provide a theoretical basis for understanding the potential toxicity of persistent HPM residues in rapeseed, with implications for pesticide environmental risk assessment and the safe production of rapeseed.

## 1. Introduction

Oilseed rape (*Brassica napus* L.) is one of the world’s most important oil crops and the primary source of edible oil in China [1]. In oilseed rape production, weed competition—particularly from grass-type weeds such as *Ambrosia artemisiifolia* L., *Alopecurus japonicus* L., and *Poa annua* L.—constitutes a major constraint on yield and quality [2,3]. Haloxyfop-P-methyl (HPM) is a widely used aryloxyphenoxypropionate (AOPP) herbicide for post-emergence control of annual and perennial grass weeds in oilseed rape, soybean, cotton, and other dicotyledonous crops [4]. According to registration data from the Chinese Pesticide Information Network, HPM ranks among the herbicide active ingredients with the largest number of registered formulations for use in rapeseed fields (http://www.chinapesticide.org.cn/ (accessed on 15 June 2026)). This widespread registration and application make HPM one of the most preferred herbicides for grass weed control in these cropping systems. Its herbicidal action relies on inhibiting acetyl-CoA carboxylase (ACCase) in susceptible grasses, thereby disrupting fatty acid biosynthesis and suppressing meristematic growth [5].

HPM exhibits relatively high environmental persistence and is resistant to photochemical and thermal degradation, with demonstrated potential for bioaccumulation [6,7]. Beyond its herbicidal activity, HPM has been reported to impair development in zebrafish and estuarine crabs [8,9] and to damage spermatogenic cells in rodents [10,11], suggesting potential reproductive and non-target toxicity. Given the extensive and repeated use of HPM in oilseed rape production, herbicide residues may accumulate in harvested seeds. However, research on the residue behavior of HPM in oilseed rape and its impact on seed germination and physiology remains extremely limited.

Seed germination marks the onset of the plant life cycle and is recognized as one of the most stress-sensitive developmental stages [12,13,14]. During this process, seeds undergo a rapid transition from quiescence to active metabolism, relying entirely on stored reserves for energy and carbon skeletons until autotrophic establishment is achieved [15]. Unlike mature plants, germinating seeds lack developed root systems, photosynthetic capacity, and the full repertoire of stress-adaptation mechanisms, rendering them especially vulnerable to suboptimal conditions [14]. Consequently, any disruption to core metabolic pathways—such as fatty acid biosynthesis and energy metabolism—during this critical window may have irreversible consequences for germination success and subsequent seedling establishment [16]. Nevertheless, whether HPM residues in seeds affect this critical process, and the underlying mechanisms involved, remain unknown.

To address this knowledge gap, we designed a multi-stage investigation centered on three interconnected questions: whether HPM residues affect seed germination; whether they induce oxidative stress or membrane damage; and what molecular pathways mediate the observed effects. We first evaluated germination performance and cell morphology in seeds harvested from HPM-treated and control field areas. To determine whether any observed phenotypic differences were associated with herbicide residue accumulation, we then quantified HPM residues in the harvested seeds. To elucidate the physiological basis of the germination phenotype, we assessed oxidative stress markers and membrane integrity. Finally, to identify the underlying molecular mechanisms, we performed integrated transcriptomic, metabolomic, and lipidomic analyses using RNA-Seq and HPLC. By integrating these multi-level analyses, this study aims to provide a mechanistic understanding of how HPM residue accumulation affects seed germination, thereby filling a critical gap in the toxicological assessment of herbicides on non-target crops. The findings will provide a theoretical basis for scientifically evaluating the ecological toxicity of HPM to non-target crops and offer references for the rational use of herbicides in oilseed fields and for pesticide environmental risk assessment.

## 2. Materials and Methods

### 2.1. Plant Materials and Growth Conditions

**Figure 1:**
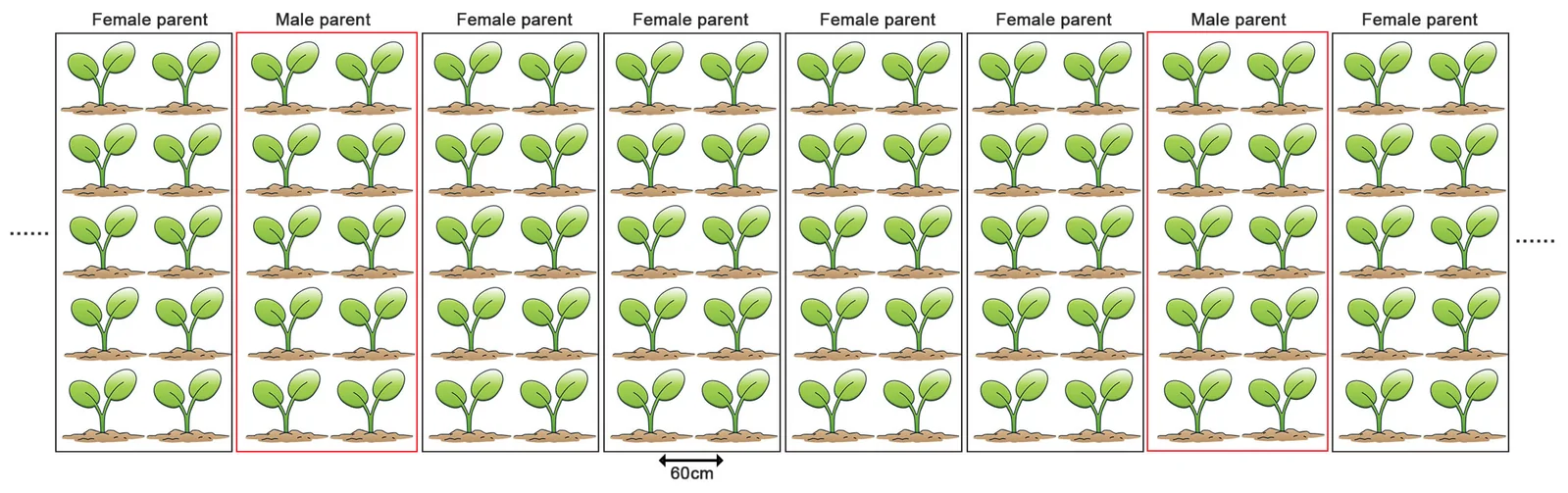
Planting mode for the hybrid seed production for Zheyouza 1510. The black box indicates the plot planted with male parent (Zheyou 19 line), and the red box indicates the plot planted with female parent (9715 × Jian6).

The hybrid rapeseed variety used in this study was Zheyouza 1510, a chemical-emasculation-based hybrid, with the female parent “9715 × Jian6” and the male parent “Zheyou 19” line. The experiment was conducted in Jiaxing, Zhejiang Province, China, during the 2023–2024 growing season. Before sowing, compound fertilizer (15-15-15, N–P_2_O_5_–K_2_O) was applied at 600 kg ha^−1^. The female and male parent seeds were sown manually in two adjacent fields on 25 September 2023, at rates of 60 kg ha^−1^ and 15 kg ha^−1^, respectively. No additional management was applied during the seedling stage. At 40 days after sowing, seedlings were transplanted to the production field. Each plot was 1.2 m wide, with two rows planted per plot at a row spacing of 60 cm. The planting pattern consisted of four plots of female parent alternating with one plot of male parent (Figure 1). The total production field covered 6.6 ha, with the previous crop being rice. Before transplanting, slow-release fertilizer (25-7-8, N–P_2_O_5_–K_2_O) was applied as a base fertilizer at 1500 kg ha^−1^ (Hubei Yishizhuang Agricultural Technology Co., Ltd., Yichang, Hubei, China). At the budding stage, a top dressing of urea was applied at 150 kg ha^−1^. Irrigation, disease, and pest management followed standard local practices for rapeseed seed production.

At the five-leaf stage of the rapeseed plants, the field was evenly divided into two areas: one was treated with Haloxyfop-P-methyl (HPM) at a rate of 81 g a.i. ha^−1^ (active ingredient), applied as the commercial formulation HPM 108 g L^−1^ EC (emulsifiable concentrate), using an unmanned aerial vehicle (UAV) for spray application. The control area received no herbicide treatment. The spray volume was 225 L ha^−1^, and the application was calibrated to ensure uniform coverage across the treated area. The application was performed under suitable weather conditions and relative humidity. No additional weed control measures were applied in either area during the growing season.

To produce hybrid seeds, a chemical hybridizing agent (CHA) was applied to the female parent. The CHA was kindly provided by Prof. Zhoubo Guan (Hybrid Rapeseed Research Center of Shaanxi Province). The reagent was prepared by mixing 240 mL of mother liquor with 225 L of water and was sprayed manually onto the female parent at two time points: (i) when the bud length reached approximately 1 mm and the plant height was about 20 cm (first application), and (ii) 10 days later (second application). To prevent cross-contamination, a cardboard barrier was used to separate female and male parent plots during spraying. At the end of flowering, male parent plants were removed to prevent seed mixing. After physiological maturity, seeds were harvested from the female parent plots using standard commercial procedures. All other agronomic practices followed conventional rapeseed seed production protocols. The harvested seeds from both the HPM-treated (HP) and control (CK) areas were used for subsequent germination assays and multi-omics analyses.

### 2.2. Seed Germination Assay

Seeds harvested from both HPM-treated and control field areas in 2024 were dried at 37 °C for 14 days after physiological maturity and then stored at room temperature (20–25 °C) in sealed containers with silica gel desiccant until use. All germination experiments were conducted within 2 months of harvest to minimize the effects of seed aging.

For each treatment, three independent biological replicates were performed, each consisting of 200 mature seeds randomly selected from the pooled harvest of each field area. Before germination, seeds were surface-sterilized by immersion in 70% ethanol for 1 min, followed by 1% sodium hypochlorite for 15 min with gentle shaking. Seeds were then rinsed five times with sterile distilled water and placed on 9-cm-diameter Petri dishes containing three layers of moist filter paper (moistened with 5 mL of sterile water). The dishes were incubated in a growth chamber at 25 ± 1 °C under a 16-h light/8-h dark photoperiod (light intensity: 10,000 μmol m^−2^ s^−1^). Germination was defined as visible radicle emergence (≥2 mm). Germinated seeds were counted every 24 h for a period of 7 days.

### 2.3. Transmission Electron Microscopy (TEM) Observation

For transmission electron microscopy (TEM) observation, mature dry rapeseed seeds from HPM-treated and control groups were directly immersed in 2.5% glutaraldehyde in 0.1 M phosphate buffer (pH 7.2) and fixed overnight at 4 °C. After washing three times with the same buffer, samples were post-fixed in 1% osmium tetroxide for 2 h at room temperature, dehydrated through a graded ethanol series (30%, 50%, 70%, 80%, 90%, 95%, and 100%; 15 min each), and embedded in Spurr‘s resin (or Epon 812). Ultrathin sections (70–90 nm) were cut using an ultramicrotome (Leica EM UC7, Leica Microsystems, Vienna, Austria), stained with 2% uranyl acetate for 15 min and 0.3% lead citrate for 5 min, and observed under a transmission electron microscope (Hitachi HT7800, Tokyo, Japan). At least 3 sections per sample were examined, and representative images were captured for each treatment. The procedure was adapted from Li et al. [17].

### 2.4. Relative Electrical Conductivity Measurement

To determine the integrity of the seed cell membrane, the relative electrolyte leakage (EL) was measured. Briefly, clean rapeseed seeds (1 g) were immersed in tubes with 20 mL of distilled water. The test tubes were incubated in a shaker at room temperature for 24 h and were assayed for initial conductivity (EC_1_). Subsequently, these tubes were boiled for 20 min. After cooling at room temperature, the second conductivity (EC_2_) was measured. Twenty mL of distilled water without tissues was assayed for EC_01_ and EC_02_ as the controls of EC_1_ and EC_2_, respectively. The relative EL value was calculated using the following formula:

EC (%) = (EC_1_ − EC_01_)/(EC_2_ − EC_02_) × 100%

### 2.5. Determination of HPM

HPM residues in mature rapeseed seeds were determined by Qingdao Huace Testing Technology Co., Ltd. (Qingdao, Shandong, China), a testing laboratory accredited under CMA (China Metrology Accreditation, No. 241520340452) and the CNAS (China National Accreditation Service, No. L4157), in compliance with ISO/IEC 17025:2017. The analysis was performed following the national standard method GB/T 20770-2008 (“Determination of pesticide residues in cereals and oil seeds—GC/MS method”), with minor modifications optimized by the testing laboratory to improve sensitivity and specificity for Haloxyfop-P-methyl detection in oilseed matrices. The detailed extraction, clean-up, and instrumental parameters are proprietary to the commercial laboratory and are not disclosed to clients under their data confidentiality policy. The flow rate of the mobile phase was 0.4 mL min^−1^. A total of 10 μL extract was injected into the LC-MS/MS system using the autosampler. 

### 2.6. Determination of Antioxidative Stress

Seeds harvested from HPM-treated and control field areas were germinated under the conditions described in Section 2.2. At 12 h and 24 h post-imbibition, seeds from each treatment were collected as three independent biological replicates (each replicate consisting of approximately 0.1 g seeds pooled from a single germination dish), immediately frozen in liquid nitrogen, and stored at −80 °C until analysis.

The activities of superoxide dismutase (SOD) and peroxidase (POD) were measured using commercial assay kits (Nanjing Jiancheng Bioengineering Institute, Nanjing, China; kit Nos SOD: A001-1-2; POD: A084-3-1) according to the manufacturer’s protocols. Hydrogen peroxide (H_2_O_2_) content was measured using a commercial kit (Nanjing Jiancheng Bioengineering Institute, kit No. A064-1-1). Malondialdehyde (MDA) content, an indicator of lipid peroxidation, was determined using a commercial kit (Nanjing Jiancheng Bioengineering Institute, kit No. A003-1-2). Reduced glutathione (GSH) content was measured using a commercial kit (Suzhou Comin Biotechnology Co., Ltd., Suzhou, China, kit No. GSH-1-W) according to the manufacturer’s protocol. Oxidized glutathione (GSSG) content was measured using a commercial kit (Suzhou Comin Biotechnology Co., Ltd., Suzhou, China, kit No. GSSG-1-W) according to the manufacturer’s protocol. Reduced ascorbic acid (AsA) content was measured using a commercial kit (Suzhou Comin Biotechnology Co., Ltd., Suzhou, China) according to the manufacturer’s protocol.

All measurements were performed with three technical replicates for each biological replicate. Data were analyzed using independent-sample *t*-tests (two-tailed) to compare HPM-treated and control groups at each time point. Statistical significance was set at *p* < 0.05. All values are reported as mean ± standard deviation (SD).

### 2.7. Transcriptome Analysis

Seeds harvested from HPM-treated and control field areas were germinated under the conditions described in Section 2.2. At 12 h and 24 h post-imbibition, seeds from each treatment were collected from three independently prepared germination dishes (approximately 0.1 g per replicate), pooled to form three independent mixed-sample libraries per time point and treatment, immediately frozen in liquid nitrogen, and stored at −80 °C until RNA extraction.

Total RNA was extracted using TRIzol reagent (Invitrogen, Carlsbad, CA, USA) according to the manufacturer’s instructions, and the RNA was used for library construction and sequencing. The RNA quantity, quality, and integrity were evaluated by a Nanodrop-2000 (Thermo Fisher Scientific, Wilmington, DE, USA) and 1% agarose gel electrophoresis, respectively. An Illumina NovaSeq 6000 sequencing platform constructed the sequencing library of the high-quality RNA samples (total ≥ 1 μg, concentration ≥ 35 ng/μL). After quantification using TBS380, the paired-end libraries were sequenced by Tepo Bio-technology Co., Ltd. (Nanjing, China) on the Illumina NovaSeq 6000 platform with a 2 × 150 bp paired-end configuration. Reads were cleaned and aligned using the HISAT2 [18] method. BAM files were generated using SAMtools (1.11) [19]. The mapped data were then processed using the Cufflinks package (2.2.1) [20]; gene read counts and FPKM matrices were generated using cuffquant, and DEGs were identified using cuffdiff. DEGs with a |log_2_FC| value ≥ 1 and a false discovery rate (FDR) < 0.05 were considered statistically significant. There were three biological replicates for each mixed sample used for RNA-seq analysis. Gene expression levels were displayed using the R package (4.3.0) (https://www.r-project.org/, accessed on 5 June 2026) heatmap. The raw RNA-sequencing data (FASTQ files) have been submitted to the NCBI Sequence Read Archive (SRA) and are accessible under BioProject accession number [PRJNA1474600].

### 2.8. Non-Targeted Metabolome Analysis

Seeds harvested from HPM-treated and control field areas were germinated under the conditions described in Section 2.2. At 12 h and 24 h post-imbibition, seeds from each treatment were collected from three independently prepared germination dishes (approximately 0.1 g per replicate), pooled to form three independent mixed-sample libraries per time point and treatment, immediately frozen in liquid nitrogen, and stored at −80 °C until metabolite extraction. Metabolites were extracted using the method described by Pushpa [21]. Non-targeted metabolome analysis was conducted using the Dionex UltiMate 3000 ultra-performance liquid chromatography (UPLC) system coupled with a High-Resolution Q-Exactive Orbitrap mass spectrometer (Thermo Scientific, San Jose, CA, USA) equipped with a heated electrospray ionization (HESI) probe. A Waters BEH C18 (100  ×  2.1 mm) column was used to separate metabolites. Mobile phase A was composed of 0.1% formic acid in LC–MS grade water, and mobile phase B was 0.1% formic acid in acetonitrile. Data were acquired in polarity-switching mode with positive and negative ionization using electrospray ionization (ESI), as described by Gunnaiah and Kushalappa. Xcalibur RAW files were converted to mzXML files using the MS converter. MZmine2 software was used to process the raw data [22]. Peak detection and baseline correction were carried out according to a protocol described by Vijayaraghavareddy [23].

### 2.9. Non-Targeted Lipidomic Analysis

#### Metabolites Extraction

Seeds harvested from HPM-treated and control field areas were germinated under the conditions described in Section 2.2. At 12 h and 24 h post-imbibition, seeds from each treatment were collected from three independently prepared germination dishes (approximately 0.1 g per replicate), pooled to form three independent mixed-sample libraries per time point and treatment, immediately frozen in liquid nitrogen, and stored at −80 °C until lipid extraction.

The LC/MS system for lipidomics analysis is composed of a Waters UPLC Acquity I-Class PLUS ultra-high-performance liquid tandem Waters UPLC Xevo G2-XS QTof high-resolution mass spectrometer. The column used was a Waters Acquity UPLC CSH C18 column (1.7 um, 2.1 × 100 mm). Positive ion mode: mobile phase A: 60% acetonitrile aqueous solution, 10 mM ammonium acetate, 0.1% formic acid; mobile phase B: 90% isopropanol–acetonitrile solution, 10 mM ammonium acetate, 0.1% formic acid. Negative ion mode: mobile phase A: 60% acetonitrile aqueous solution, 10 mM ammonium acetate, 0.1% formic acid; mobile phase B: 90% isopropanol–acetonitrile solution, 10 mM ammonium acetate, 0.1% formic acid. Injection volume: 5 μL, flow rate: 0.4 mL/min, column temperature: 55 degrees Celsius.

Principal component analysis and Spearman correlation analysis were used to judge the repeatability of the samples within groups and the quality control samples. Classification and pathway information for the identified compounds were obtained from the KEGG, HMDB, and LipidMaps databases. According to the grouping information, to calculate and compare the difference multiples, a *t*-test was used to calculate the *p*-value of each compound. The R language package ropls was used to perform OPLS-DA modeling, and 200 permutation tests were performed to verify the reliability of the model. The VIP value of the model was calculated using multiple cross-validation. The method of combining the difference multiple, the *p*-value, and the VIP value of the OPLS-DA model was adopted to screen the differential metabolites. The screening criteria were FC > 2, *p*-value < 0.01, and VIP > 1.

### 2.10. Statistical Analysis

All statistical analyses were performed using GraphPad Prism 8 (GraphPad Software, San Diego, CA, USA) and R software (version 4.3.0). All data are expressed as mean ± standard deviation (SD) from at least three independent biological replicates. Pairwise comparisons between two groups (e.g., control vs. HPM-treated at the same time point) were conducted using a two-tailed Student’s *t*-test. For multiple-group comparisons (e.g., across different time points within the same treatment), one-way ANOVA followed by Tukey’s honest significant difference (HSD) post-hoc test was applied. The GLM analysis was performed using the glm (4.3.0) function in R with the ‘car’ package for ANOVA-type Wald chi-square tests. Statistical significance was set at * *p* < 0.05, ** *p* < 0.01, and *** *p* < 0.001.

## 3. Results

### 3.1. Germination Percentage and Seed Ultrastructure in HPM-Treated and Control Rapeseed

**Figure 2:**
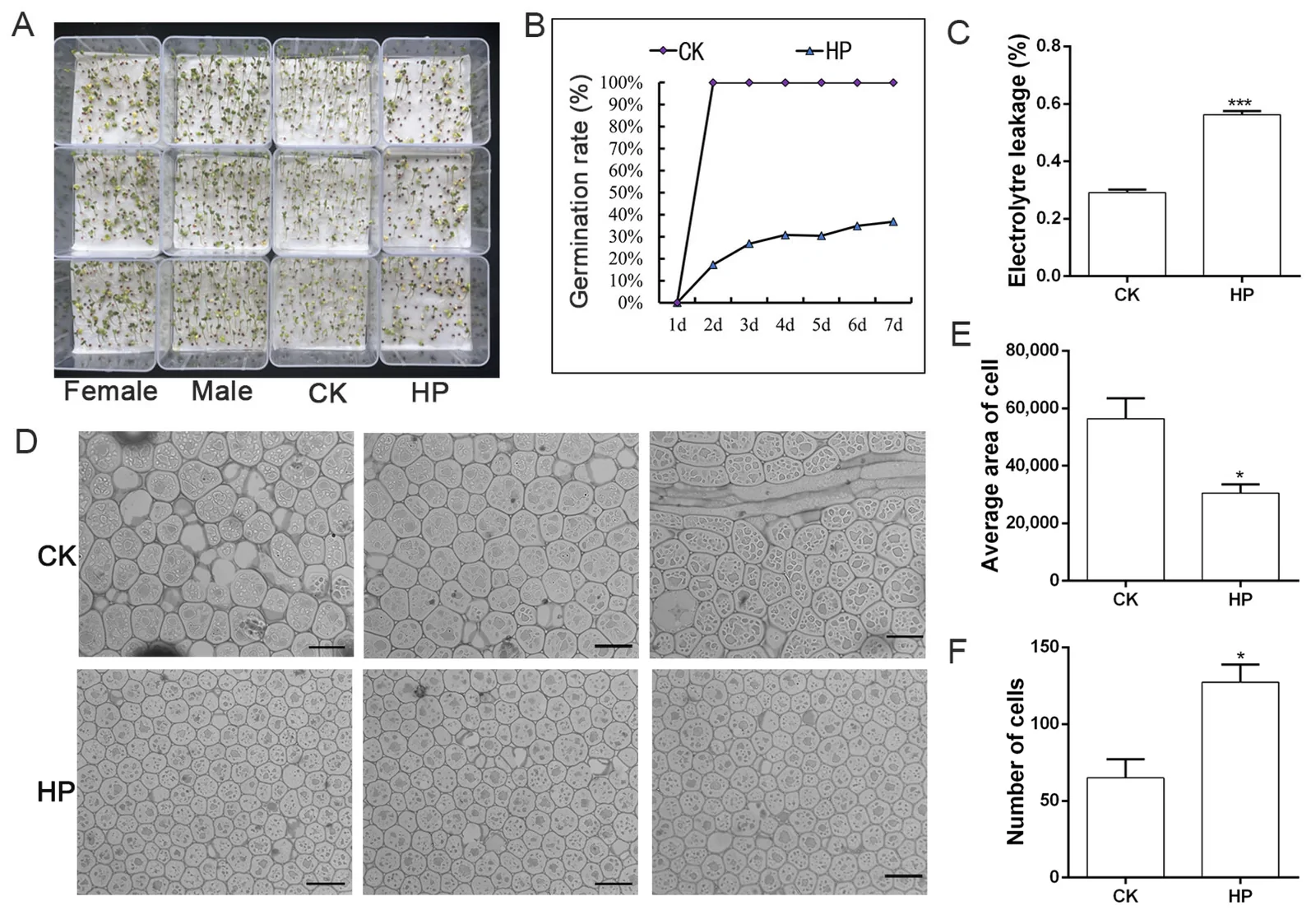
Effect of HPM on seed germination, membrane integrity, and cell morphology of rapeseed. (**A**) Phenotypes of seed germination for female, male, CK, and HP. (**B**) Seed germination rates for CK and HP. (**C**) Relative electrical conductivity (REC) of dry seeds from CK and HP groups. (**D**) Observation of cell morphology of CK and HP. Bar = 2 μm. (**E**) Statistical analysis of the average area of CK and HP cells. (**F**) Counting of cell numbers under the same perspective for CK and HP. CK: harvested seeds were not applied with HPM; HP: harvested seeds were applied with HPM. Data are expressed as mean ± SD of three biological replicates (*n* = 3), and each biological replicate contains at least three technical replicates. Statistical significance was determined by one-way ANOVA combined with Tukey’s test (* *p* < 0.05 and *** *p* < 0.001).

To characterize the quality of seeds harvested from HPM-treated and control field plots, we first compared their germination performance and cell morphology. The final germination percentage of HPM-treated seeds was 36.5%, which was significantly lower than that of the control group (100%) (Figure 2A,B). Scanning electron microscopy (SEM) observations revealed that HPM-treated seeds exhibited altered cell morphology relative to the control, characterized by more densely packed cells and reduced cell size (Figure 2D–F). To assess whether membrane integrity was compromised, we measured the relative electrical conductivity (REC) of dry seeds. The REC of HPM-treated seeds was significantly higher than that of the control (Figure 2C), indicating that cell membrane integrity had already been impaired in dry seeds prior to germination. Collectively, these observations demonstrate that seeds derived from HPM-treated plants differed from control seeds with respect to germination percentage, cell morphology, and membrane integrity.

### 3.2. HPM Residue Is Detectable in Seeds from the Treated Field

**Figure 3:**
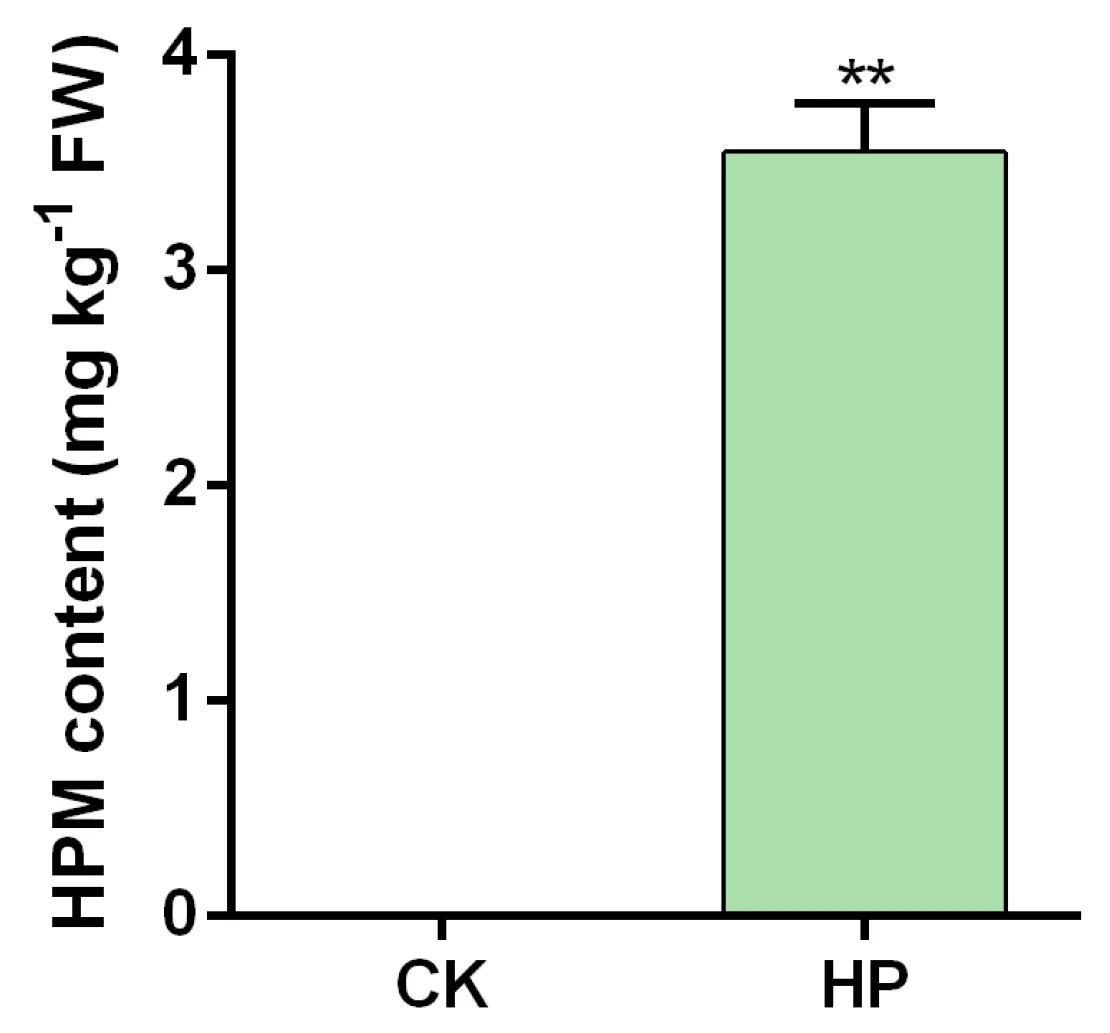
HPM residue concentration in rapeseed seeds. HPM residues were detected in seeds harvested from HPM-treated (HP) and control (CK) field areas. Data are expressed as mean ± SD of three biological replicates (*n* = 3), with each biological replicate containing at least three technical replicates. Statistical significance was determined by Student‘s *t*-test (** *p* < 0.01).

To determine whether the observed phenotypic differences were associated with herbicide residue accumulation, we quantified HPM residues in the harvested seeds. No detectable HPM residues were found in control rapeseed, whereas the HPM-treated group contained a high residue level of 3.36 mg kg^−1^ (Figure 3). Furthermore, this value exceeded the maximum residue limit (MRL) of 3 mg kg^−1^ established for haloxyfop in rapeseed by the Chinese national food safety standard (GB 2763-2021) [24]. Collectively, these results indicate that seeds derived from HPM-treated plots contained quantifiable HPM residues above the regulatory MRL, while control seeds remained residue-free.

### 3.3. Oxidative Damage Induced by HPM in Rapeseed

**Figure 4:**
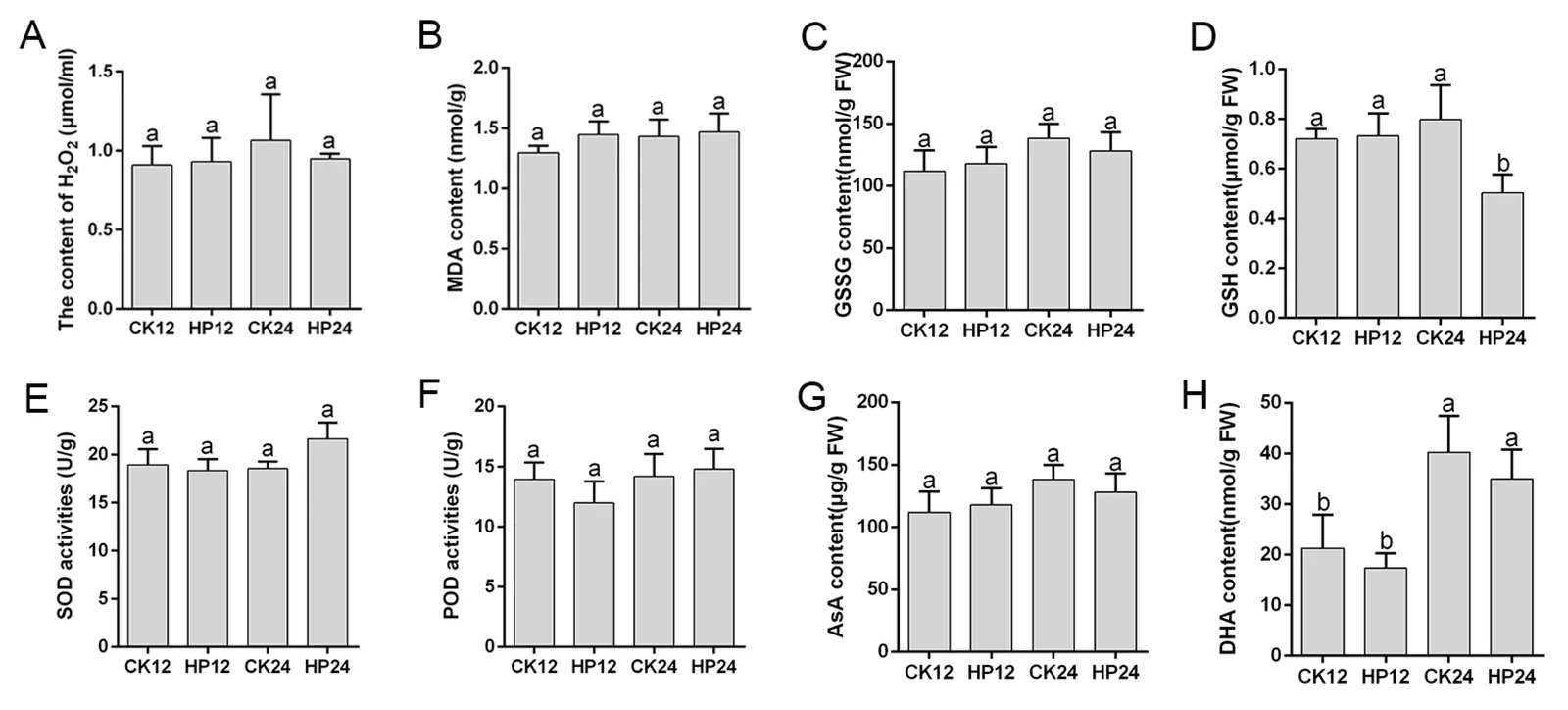
Analysis of oxidative stress markers and antioxidant parameters in germinating rapeseed seeds. (**A**) Hydrogen peroxide (H_2_O_2_) content. (**B**) Malondialdehyde (MDA) content. (**C**) Oxidized glutathione (GSSG) content. (**D**) Reduced glutathione (GSH) content. (**E**) Superoxide dismutase (SOD) activity. (**F**) Peroxidase (POD) activity. (**G**) Reduced ascorbic acid (AsA) content. (**H**) Dehydroascorbic acid (DHA) content. CK12: seeds harvested from control field area, sampled at 12 h post-imbibition. HP12: seeds harvested from HPM-treated field area, sampled at 12 h post-imbibition. CK24: seeds harvested from control field area, sampled at 24 h post-imbibition. HP24: seeds harvested from HPM-treated field area, sampled at 24 h post-imbibition. Data are expressed as mean ± SD of three biological replicates (*n* = 3), with each biological replicate containing at least three technical replicates. Statistical significance was determined by one-way ANOVA combined with Tukey’s test. Different letters indicate significant differences between groups (*p* < 0.05).

To explore whether oxidative damage could account for the reduced germination and impaired membrane integrity observed in HPM-treated seeds, we measured oxidative stress markers during germination (12 h and 24 h post-imbibition). We quantified the contents of hydrogen peroxide (H_2_O_2_) and malondialdehyde (MDA) as indicators of oxidative damage, and determined the activities of superoxide dismutase (SOD) and peroxidase (POD) as representative antioxidant enzymes. In addition, we measured the contents of reduced glutathione (GSH), oxidized glutathione (GSSG), reduced ascorbic acid (AsA), and dehydroascorbic acid (DHA) to assess the non-enzymatic antioxidant system. The results revealed no significant differences in any of these parameters between the HPM-treated and control groups at either 12 h or 24 h (Figure 4A–H). These findings indicate that HPM residues in seeds did not induce detectable oxidative stress during the early germination stages. Consistently, the observed reductions in germination percentage and membrane integrity were not associated with oxidative damage under our experimental conditions.

### 3.4. Fatty Acid Biosynthesis Pathways Are Downregulated at the Transcriptional Level

**Figure 5:**
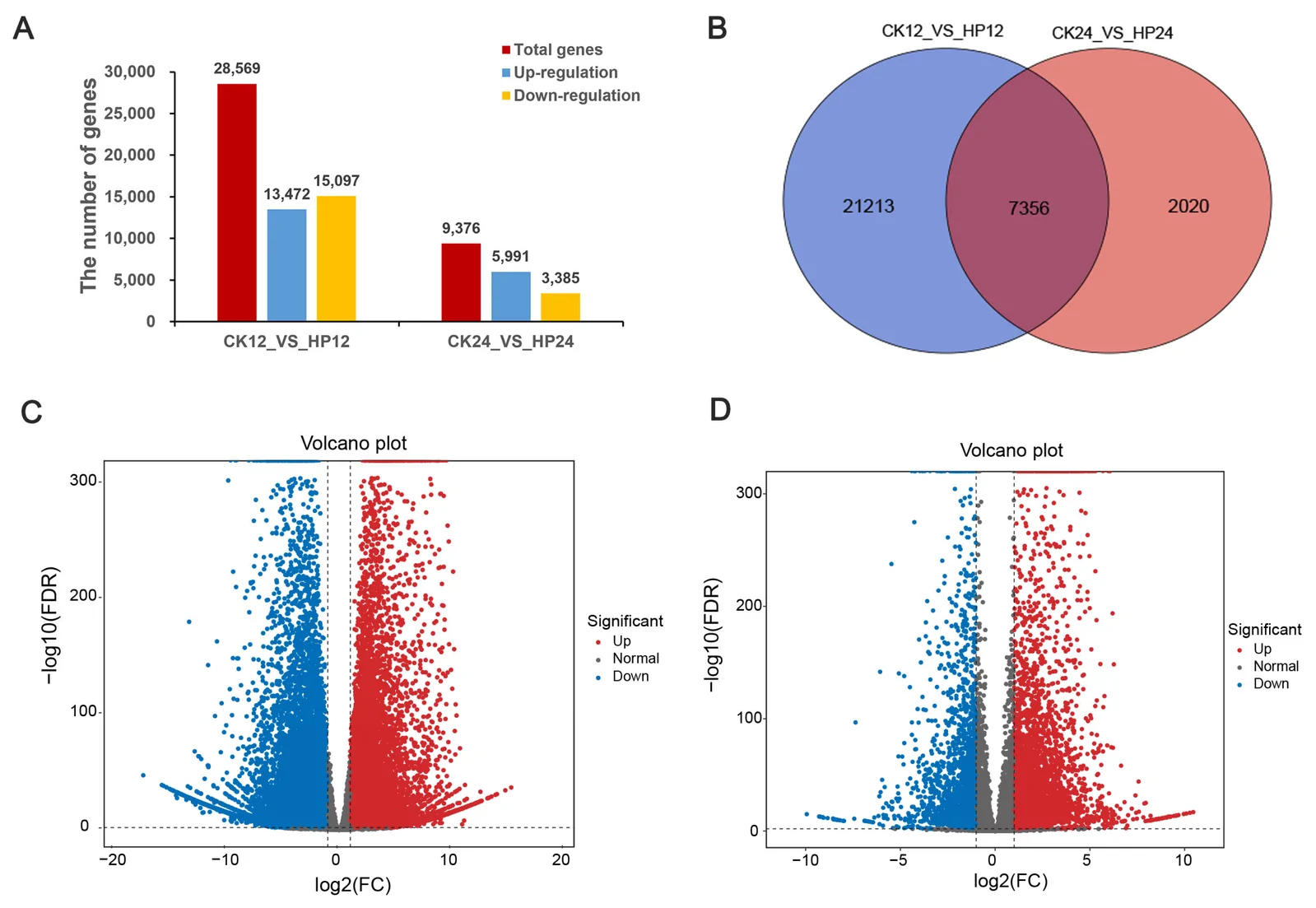
Landscape of differentially expressed genes (DEGs) in rapeseed seeds in response to HPM residue accumulation. (**A**) Number of significantly upregulated and downregulated genes in HPM-treated seeds compared to controls at 12 h and 24 h post-imbibition. (**B**) Venn diagram analysis displaying DEGs between the two time points. (**C**) Volcano plot of DEGs at 12 h post-imbibition. (**D**) Volcano plot of DEGs at 24 h post-imbibition. CK: control seeds; HP: HPM-treated seeds. DEGs were defined as |log_2_FC| ≥ 1 and false discovery rate (FDR) < 0.05. Data are from three independently prepared mixed-sample libraries per time point.

To identify the molecular pathways underlying the reduced germination and impaired membrane integrity observed in HPM-treated seeds, we performed transcriptomic profiling of germinating seeds at 12 h and 24 h post-imbibition using RNA-seq. All RNA samples had RIN values ≥ 8.0, confirming their suitability for cDNA library construction (Table S1). RNA-seq generated an average of 49.6 million clean reads per sample, with a mean alignment rate of 90.86% to the rapeseed reference genome (Table S2), indicating robust data quality for downstream differential expression analysis. Principal component analysis and sample clustering revealed that the global transcriptional profiles of HPM-treated seeds differed from those of controls at both time points (Figure S1). Volcano plots further illustrated the extent of these differences (Figure 5C,D). At 12 h post-imbibition, HPM-treated seeds exhibited 13,472 upregulated and 15,097 downregulated differentially expressed genes (DEGs) compared with controls (Figure 5A and Table S3). At 24 h, the number of DEGs was lower, with 5991 upregulated and 3385 downregulated genes (Figure 5A and Table S4). The expression pattern of differentially expressed genes (DEGs) in each cDNA library was investigated by Venn diagram analysis (Figure 5B).

**Figure 6:**
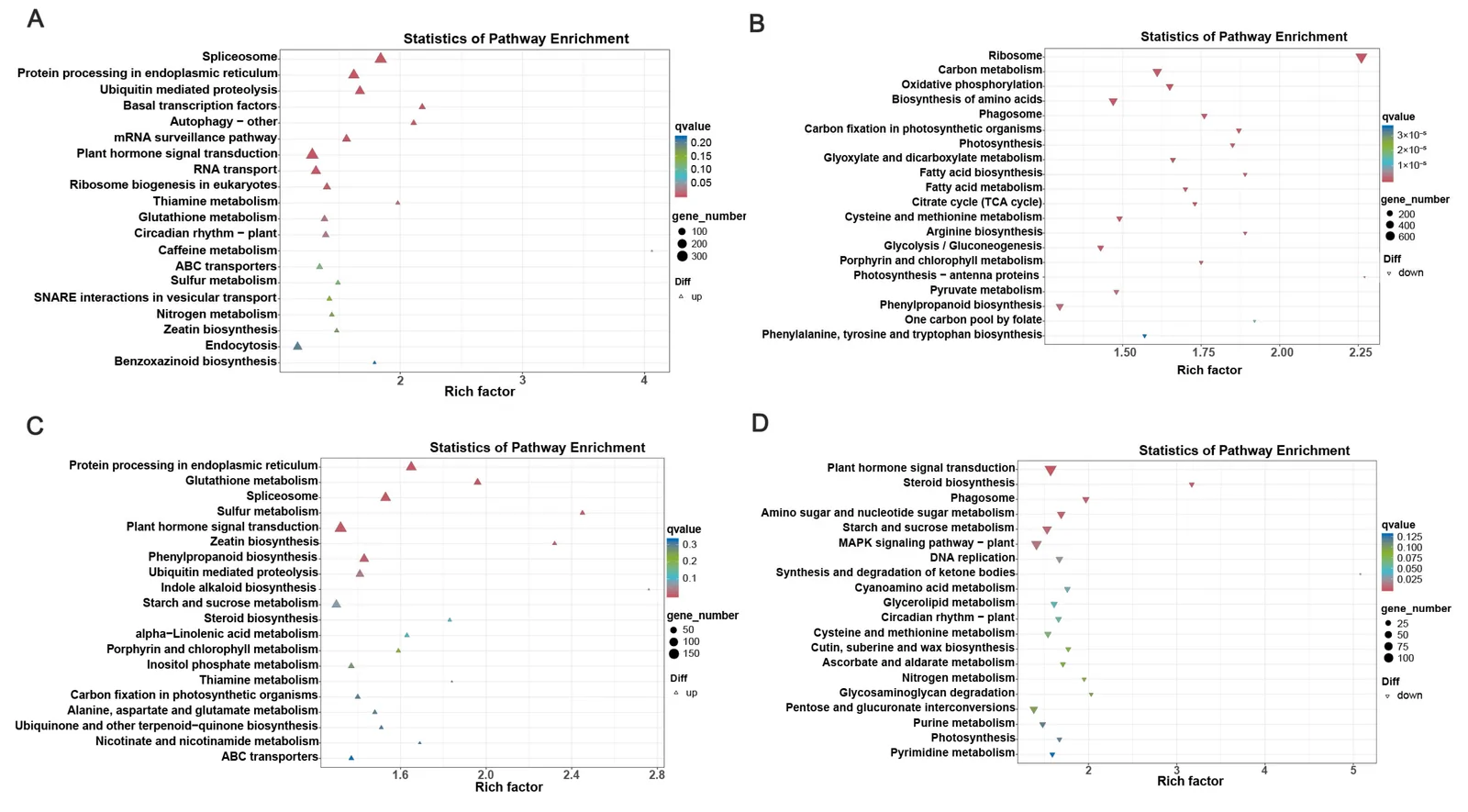
KEGG pathway enrichment analysis of differentially expressed metabolites (DEMs) in HPM-treated versus control seeds at 12 h and 24 h post-imbibition. (**A**) Enriched pathways among upregulated metabolites at 12 h. (**B**) Enriched pathways among downregulated metabolites at 12 h. (**C**) Enriched pathways among upregulated metabolites at 24 h. (**D**) Enriched pathways among downregulated metabolites at 24 h. Only the most significantly enriched pathways are shown (*p* < 0.05). The size of each triangle represents the number of metabolites enriched in the pathway, and the color indicates the significance level (*p*-value). CK: control seeds; HP: HPM-treated seeds. Data are from three independently prepared mixed-sample libraries per time point.

KEGG enrichment analysis revealed that upregulated DEGs were predominantly enriched in pathways related to the spliceosome, protein processing in the endoplasmic reticulum, plant hormone signal transduction, glutathione metabolism, and nitrogen metabolism (Figure 6A,C). By contrast, downregulated DEGs were mainly associated with carbon metabolism, oxidative phosphorylation, fatty acid biosynthesis and metabolism, and carbon fixation in photosynthetic organisms (Figure 6B,D). Among the downregulated genes, key enzymes involved in glycolysis (e.g., HXK1, HXK2, GAPDH), the tricarboxylic acid (TCA) cycle (e.g., mMDH1), and fatty acid synthesis (e.g., KAS1, FAD3) were significantly downregulated in HPM-treated seeds. In contrast, genes involved in pre-mRNA splicing (SUS2), cell division control (CDC48A), and glutamate metabolism (GDH2) were significantly upregulated (Table S10).

**Figure 7:**
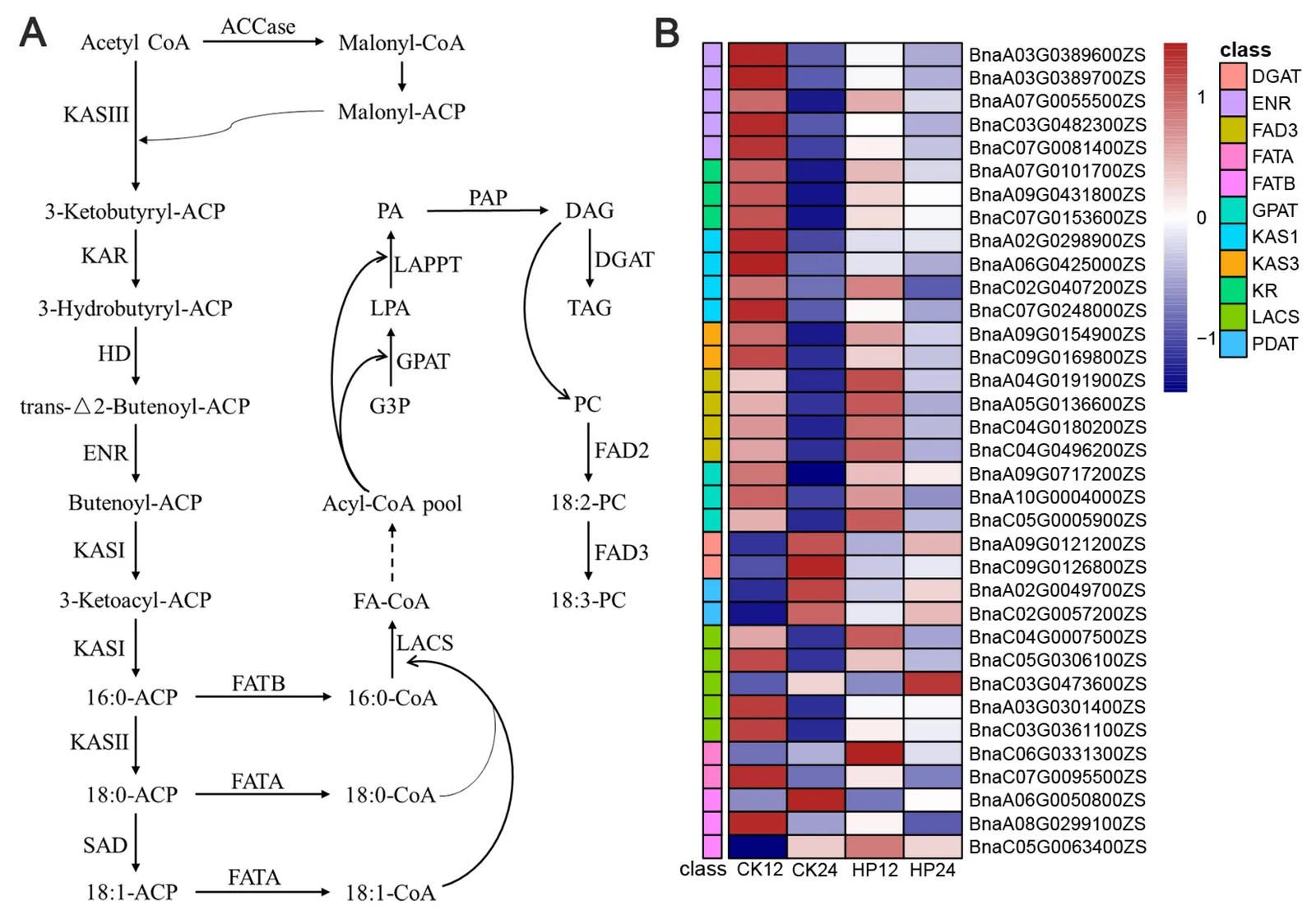
Fatty acid biosynthesis pathway and expression patterns of related genes in HPM-treated versus control seeds. (**A**) Schematic representation of the fatty acid biosynthesis pathway in rapeseed. (**B**) Heatmap showing the expression levels of key genes involved in fatty acid biosynthesis at 12 h and 24 h post-imbibition. Red indicates upregulated expression; blue indicates downregulated expression. CK: control seeds; HP: HPM-treated seeds. Data are from three independently prepared mixed-sample libraries per time point.

To validate the transcriptional downregulation of fatty acid biosynthesis, we examined the expression patterns of key genes involved in this pathway. A schematic representation of the fatty acid biosynthesis pathway in rapeseed is shown in Figure 7A, and the corresponding heatmap (Figure 7B) revealed that genes encoding core enzymes—including KAS1, KAS1, ENR, LACS, GPAT, and FAD3—were consistently downregulated in HPM-treated seeds at both 12 h and 24 h post-imbibition relative to controls. These findings are consistent with the KEGG enrichment results (Figure 6B) and collectively support the association between HPM residue accumulation and the suppression of fatty acid biosynthesis during seed germination.

Collectively, these transcriptomic data indicate that HPM residue accumulation was associated with the downregulation of pathways central to energy metabolism and fatty acid biosynthesis, alongside the upregulation of stress-related and regulatory pathways. The repression of carbon metabolism, oxidative phosphorylation, and fatty acid synthesis—as evident from both the KEGG enrichment analysis and the expression patterns of key genes (KAS1, KAS1, ENR, LACS, GPAT, and FAD3) shown in the heatmap (Figure 7) —is consistent with the observed reduction in germination percentage. These transcriptional changes suggest that impaired energy and lipid metabolism may contribute to the germination phenotype of HPM-treated seeds.

### 3.5. Metabolomic Profiles Are Altered in HPM-Treated Seeds

**Figure 8:**
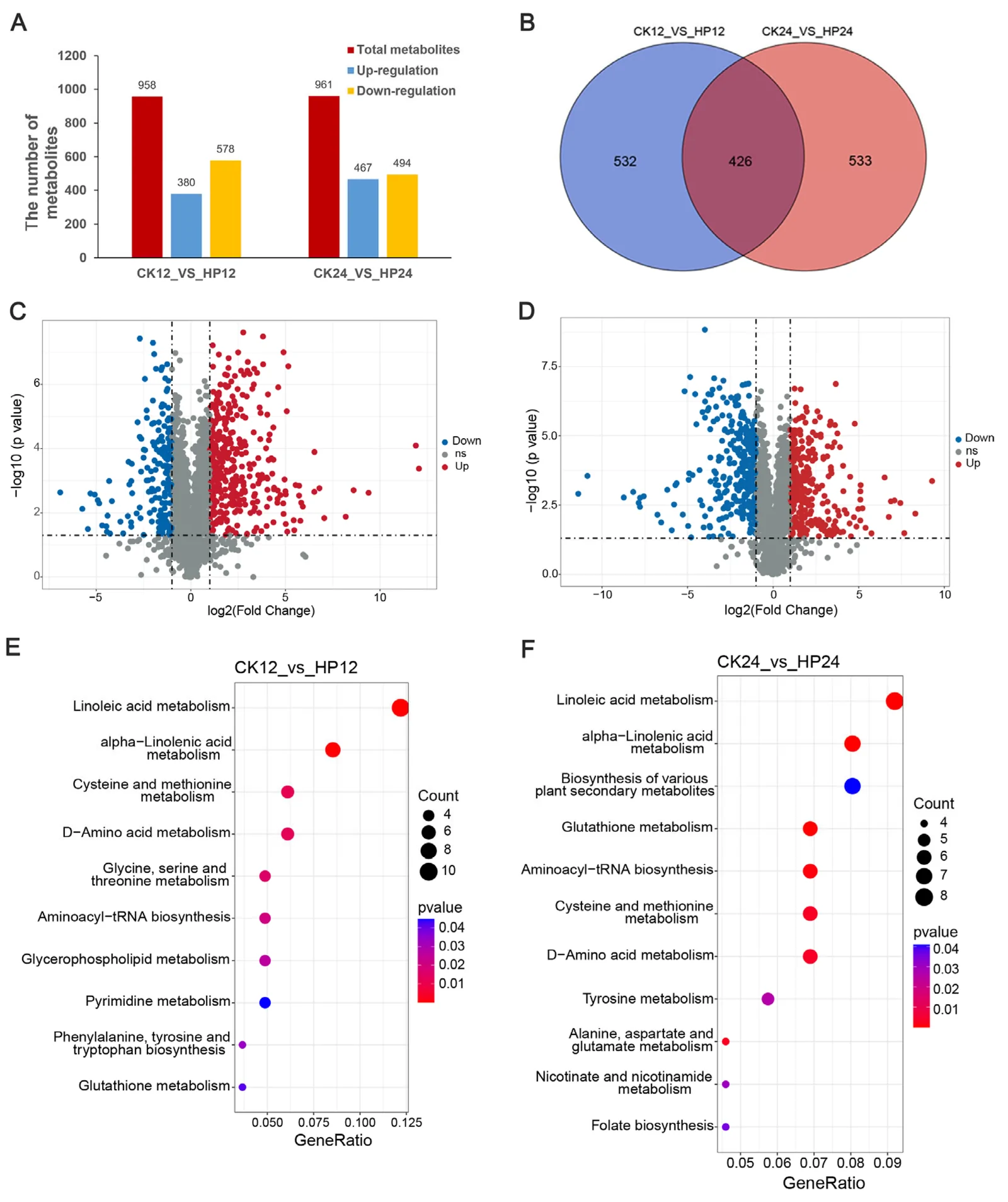
Landscape of DEMs and functional enrichment analysis of rapeseed in response to HPM stress from metabolomics data. (**A**) The number of significantly upregulated and downregulated metabolites among the control and HPM-treated rapeseed. (**B**) Venn diagram analysis displaying DEMs of seeds from each group after germination. (**C**) Volcano plot of DEMs from seeds germinated for 12 h. Dashed lines indicate the thresholds for statistical significance (p<0.05) and fold change (log_2_(FC) > 1). (**D**) Volcano plot of DEMs from seeds germinated for 24 h. Dashed lines indicate the thresholds for statistical significance (p<0.05) and fold change (log_2_(FC) > 1). (**E**) KEGG pathway analysis for the upregulated metabolites from seeds germinated for 12 h. (**F**) KEGG pathway analysis for the down-regulated metabolites from seeds germinated for 12 h.

To determine whether the transcriptional changes described above were reflected at the metabolite level, we performed untargeted metabolomic profiling of germinating seeds at 12 h and 24 h post-imbibition. Principal component analysis and sample clustering revealed that the metabolic profiles of HPM-treated seeds differed from those of controls at both time points (Figure S2). Volcano plots further demonstrate significant differences in metabolite accumulation between HPM-treated and control seeds (Figure 8C,D).

At 12 h post-imbibition, HPM-treated seeds exhibited 380 upregulated and 578 downregulated differentially expressed metabolites (DEMs) compared with controls (Figure 8A; Table S5). At 24 h, 467 upregulated and 494 downregulated DEMs were detected (Figure 8A; Table S6). The expression pattern of DEMs in each cDNA library was investigated by Venn diagram analysis (Figure 8B).

KEGG pathway enrichment analysis revealed that the DEMs were predominantly enriched in linoleic acid metabolism, α-linolenic acid metabolism, glycerophospholipid metabolism, and amino acid metabolism (including glutathione metabolism, cysteine and methionine metabolism, D-amino acid metabolism, and glycine–serine–threonine metabolism) (Figure 8E,F). In HPM-treated seeds, the relative levels of linolenic acid derivatives (e.g., hydroperoxy octadecatrienoic acid (HpOTrE), FA 18:3, and norlinolenic acid) were significantly increased, whereas those of glycerophospholipids (e.g., lysophosphatidic acid 18:2 (LPA 18:2), lysophosphatidylinositol 18:1 (LPI 18:1), and TriHOME) were significantly decreased compared with controls (Table S11).

Collectively, these metabolomic data indicate that HPM residue accumulation was associated with alterations in fatty acid- and amino acid-related metabolic pathways, consistent with the transcriptional downregulation of fatty acid biosynthesis genes observed above.

### 3.6. Lipidomic Profiles Are Altered in HPM-Treated Seeds

**Figure 9:**
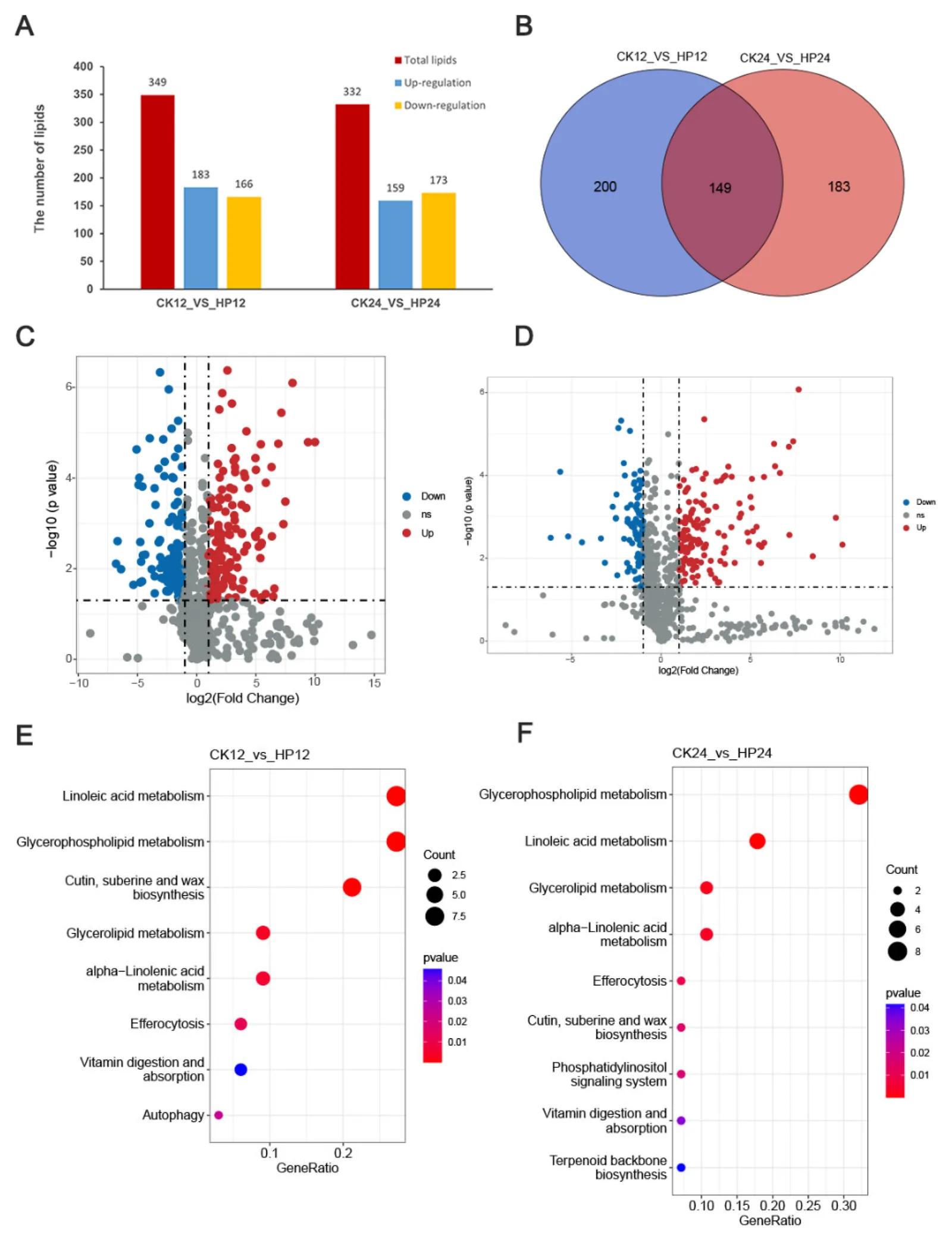
Landscape of DELs and functional enrichment analysis of rapeseed in response to HPM stress from lipidomics data. (**A**) The number of significantly upregulated and downregulated lipids among the control and HPM-treated rapeseed. (**B**) Venn diagram analysis displaying DELs of seeds from each group after germination. (**C**) Volcano plot of DELs from seeds germinated for 12 h. Dashed lines indicate the thresholds for statistical significance (p<0.05) and fold change (log_2_(FC) > 1). (**D**) Volcano plot of DELs from seeds germinated for 24 h. Dashed lines indicate the thresholds for statistical significance (p<0.05) and fold change (log_2_(FC) > 1). (**E**) KEGG pathway analysis for the upregulated lipids from seeds germinated for 12 h. (**F**) KEGG pathway analysis for the down-regulated lipids from seeds germinated for 12 h.

To investigate whether the downregulation of fatty acid biosynthesis genes and the alterations in fatty acid-related metabolites described above were reflected in lipid composition, we performed untargeted lipidomic profiling of germinating seeds at 12 h and 24 h post-imbibition. Principal component analysis and sample clustering revealed that the lipid profiles of HPM-treated seeds differed from those of controls at both time points (Figure S3). Volcano plots further demonstrate significant differences in lipid accumulation between HPM-treated and control seeds (Figure 9C,D).

At 12 h post-imbibition, HPM-treated seeds exhibited 183 upregulated and 166 downregulated differentially expressed lipids (DELs) compared with controls (Figure 9A; Table S7). At 24 h, 159 upregulated and 173 downregulated DELs were detected (Figure 9F; Table S8). The expression pattern of DELs in each cDNA library was investigated by Venn diagram analysis (Figure 9B).

KEGG pathway enrichment analysis revealed that the DELs were predominantly enriched in linoleic acid metabolism, α-linolenic acid metabolism, glycerolipid metabolism, glycerophospholipid metabolism, and cutin, suberine, and wax biosynthesis (Figure 9E), which were largely consistent with the metabolomic findings described above. In HPM-treated seeds, the relative abundance of linoleic acid derivatives (e.g., diHODE and FA 18:2;O2) was significantly altered, whereas the relative levels of glycerolipids and glycerophospholipids (e.g., TG 20:2_20:4_22:0, PE 16:0_18:3, and PG 14:0;O-14:0;O) also showed significant changes compared with the control (Table S12).

Collectively, these lipidomic data indicate that HPM residue accumulation was associated with alterations in glycerolipid and glycerophospholipid composition, together with changes in linoleic acid and α-linolenic acid metabolism (Figure S4). These findings are consistent with the transcriptional downregulation of fatty acid biosynthesis genes and the metabolomic changes observed above, further supporting the association between HPM residue accumulation and disrupted lipid metabolism in germinating rapeseed seeds.

Collectively, these lipidomic data indicate that HPM residue accumulation was associated with substantial remodeling of the seed lipidome, characterized by reduced glycerolipids and glycerophospholipids, altered linoleic acid dynamics during germination, and increased prenol lipids (Figure S4). These changes were consistent with the downregulation of fatty acid biosynthesis genes (Section 3.4), supporting the association between HPM residue accumulation and disrupted lipid metabolism in germinating rapeseed seeds.

## 4. Discussion

### 4.1. HPM Residues in Rapeseed Seeds Are Associated with Reduced Germination and Impaired Membrane Integrity

The most striking phenotypic observation of this study was the marked reduction in germination percentage in HPM-treated seeds (36.5%) compared with the control (100%), as well as abnormal cell morphology, and compromised membrane integrity in dry seeds, as indicated by elevated relative electrical conductivity (Figure 2). These observations established the central question of our investigation: what physiological and molecular mechanisms underlie this germination failure?

HPM residue analysis confirmed the presence of the herbicide in treated seeds at 3.36 mg kg^−1^, exceeding the Chinese MRL of 3 mg kg^−1^ established under GB 2763 (Figure 3 and Figure S5). This finding is consistent with the known environmental persistence and bioaccumulation potential of HPM [25], and aligns with reports on residue risks of other ACCase-inhibiting herbicides in crops [26,27,28]. The detection of HPM residues above the regulatory limit raises concerns about the potential non-target effects of this herbicide on crop seed quality, a dimension that has received limited attention in previous risk assessments. Notably, the elevated electrical conductivity in dry seeds indicates that membrane integrity was already compromised prior to germination, suggesting that structural damage may have occurred during seed development on the mother plant rather than during the germination assay itself.

### 4.2. Oxidative Stress Was Not Detectable During Germination, but Membrane Damage May Have Originated During Seed Development

Herbicides commonly induce oxidative stress through the generation of reactive oxygen species (ROS) [29,30]. In the present study, however, the antioxidant markers H_2_O_2_, MDA, SOD, POD, GSH, GSSG, and AsA showed no significant changes in HPM-treated seeds at either 12 h or 24 h post-imbibition (Figure 4), indicating that oxidative stress was not detectable during the germination period under our experimental conditions.

This absence of oxidative response does not, however, exclude the possibility that oxidative injury occurred at an earlier developmental stage. HPM is typically applied during the rosette stage of rapeseed, when seeds are still developing on the mother plant. The elevated electrical conductivity observed in dry HPM-treated seeds—indicative of pre-existing membrane damage—supports the hypothesis that oxidative injury may have occurred during seed maturation, with the damage persisting as a structural defect into the dry seed state. The lack of oxidative markers during germination may therefore reflect the timing of sampling (12 h and 24 h), as any transient ROS burst triggered by rehydration would likely occur within minutes of imbibition, prior to our first sampling point. Future studies incorporating earlier time points (e.g., 0–6 h post-imbibition) or direct ROS imaging in developing seeds would be required to address this hypothesis.

### 4.3. HPM Residues Are Associated with Transcriptional Downregulation of Fatty Acid Biosynthesis and Energy Metabolism

The transcriptomic data revealed that genes involved in fatty acid biosynthesis (KAS1, KAS1, ENR, LACS, GPAT, and FAD3), the TCA cycle, and oxidative phosphorylation were significantly downregulated in HPM-treated seeds (Figure 7). These findings are consistent with the known mode of action of HPM as an ACCase inhibitor, which disrupts the rate-limiting step of de novo fatty acid synthesis [30,31]. However, given that *Brassica napus* possesses a heteromeric plastidic ACCase that is generally less sensitive to aryloxyphenoxypropionate herbicides, the observed transcriptional suppression may involve indirect mechanisms—such as stress-signaling pathways or hormone-mediated regulation—rather than direct target inhibition.

The downregulation of fatty acid biosynthesis genes was corroborated at the metabolite level. Metabolomic analysis revealed significant enrichment of pathways related to linoleic acid and α-linolenic acid metabolism, while lipidomics showed reduced relative abundance of glycerolipids and glycerophospholipids, alongside increased prenol lipids (Figure S4). The altered lipid composition, combined with the transcriptional suppression of energy metabolism pathways, suggests that HPM residue accumulation was associated with impaired lipid reserves and energy supply. In oilseeds, germination relies heavily on the mobilization of stored lipids via β-oxidation to provide ATP and carbon skeletons for radicle emergence [32]. Disruption of fatty acid biosynthesis and energy metabolism during this critical window may therefore compromise the energy supply required for successful germination—a prediction consistent with the observed 36.5% germination rate in HPM-treated seeds.

Consistent with previous studies demonstrating herbicide-induced activation of amino acid metabolism pathways [33,34,35,36], our metabolomic data showed significant enrichment of D-amino acid and glutathione metabolism pathways (Figure 6A). The glutathione metabolism pathway, in particular, may reflect a systemic response to oxidative conditions during seed development, as glutathione plays a crucial role in cellular protection against oxidative damage [37,38,39]. The persistence of glutathione-related gene expression during germination further supports the hypothesis that residual HPM may trigger stress responses during early imbibition, although direct evidence for an oxidative burst remains to be obtained.

### 4.4. Altered Linolenic Acid Dynamics and Sterol Deficiency May Impair Germination

Lipidomic analysis revealed a distinctive pattern of α-linolenic acid (C18:3) dynamics during germination: its relative abundance was significantly higher in HPM-treated seeds than in controls at 12 h, but decreased substantially by 24 h, whereas control seeds showed the opposite trend (Figure S4). Free α-linolenic acid has been reported to exert cytotoxic effects by inserting into lipid bilayers, disrupting membrane integrity, and activating programmed cell death signals through its role as a jasmonic acid precursor [40]. The transient increase in relative abundance of free α-linolenic acid in HPM-treated seeds at 12 h may reflect membrane lipid remodeling or the release of fatty acids from damaged membranes (Figure S4), while its subsequent decline by 24 h could be attributed to substrate depletion or a general reduction in cellular metabolic activity. Although these observations are consistent with the proposed cytotoxicity of free fatty acids, the available data do not conclusively establish a causal relationship between the accumulation of α-linolenic acid (as reflected by its relative abundance) and germination failure.

Sterols, synthesized from acetyl-CoA via the mevalonate pathway [41,42], are essential for membrane structure and cell plate formation during cytokinesis [43,44]. Transcriptomic analysis revealed downregulation of steroid biosynthesis-related genes (Figure 6B), and lipidomics confirmed a reduction in sterol relative abundance in HPM-treated seeds. Scanning electron microscopy further revealed reduced cell size and increased cell density in HPM-treated seeds, suggesting a disruption in the coordination between cell division and cell expansion. Given that reduced sterol availability may restrict new membrane formation, this could impair cell expansion and hypocotyl elongation, thereby preventing radicle emergence. This interpretation is consistent with the observed morphological abnormalities in germinating HPM-treated seeds and provides a mechanistic link between lipidome remodeling and failed germination.

### 4.5. Limitations and Implications for Pesticide Risk Assessment

Several methodological limitations should be acknowledged. First, the field experiment was conducted in a single production field divided into two areas without randomized replicated plots; therefore, the findings should be regarded as a preliminary case study requiring further validation. Second, the metabolomics and lipidomics data are based on relative quantification without absolute validation, and the reported metabolites should therefore be considered as putatively annotated (MSI Level 2). Third, oxidative stress was only measured during germination, and the hypothesis of oxidative injury during seed development remains speculative and requires direct testing. Additionally, regarding residue analysis, our quantification specifically targeted the parent ester rather than total haloxyfop residues as defined by the international residue definition, and the measured values therefore represent only a portion of the total residues.

Despite these limitations, the present study provides multi-level evidence (physiological, transcriptomic, metabolomic, and lipidomic) that HPM residue accumulation is associated with impaired germination in rapeseed through the disruption of fatty acid biosynthesis, energy metabolism, and lipid composition, rather than through detectable oxidative stress during germination. Although oxidative stress was not detected during germination, the observed lipidome remodeling—particularly the increased relative abundance of prenol lipids with antioxidant properties and the alteration of membrane lipid composition—remains relevant to the broader context of oxidative balance in plant seeds. These findings suggest that current pesticide risk assessment frameworks, which primarily focus on yield and residue limits, may underestimate the potential impact of herbicide residues on seed quality. We propose that seed germination performance and cell morphology should be considered as complementary endpoints in the toxicity assessment of herbicides applied to reproductive crops (e.g., oil crops, soybean, and peanut). The results also underscore the need for strict adherence to recommended application rates and timing for HPM in oilseed rape production to minimize residue accumulation in harvested seeds.

## 5. Conclusions

The findings of this study have clear practical implications for oilseed rape production. The detection of HPM residues above the MRL in harvested seeds, together with the observed reduction in germination percentage and abnormal seed cell morphology, indicates that the perceived safety of HPM for dicotyledonous crops may be overstated when the herbicide is applied at elevated rates. Strict adherence to recommended application rates and timing is therefore essential, not only to safeguard current-season yield but also to ensure seed quality for subsequent planting.

Current pesticide risk assessment frameworks primarily focus on yield impacts and residue limits in relation to dietary safety. Our results suggest that seed germination performance and seed cell morphology should be considered as complementary toxicological endpoints for herbicides applied to oil crops, as reliance on yield and residue data alone may underestimate the effects of residue accumulation on seed quality. Based on these findings, we propose the following recommendations: (1) reinforcement of label instructions to clearly communicate the potential risks of HPM to oilseed rape and to discourage over-application during the crop growth period; (2) strict adherence to recommended dosages and application windows in oilseed rape–grass crop rotation systems to minimize soil and seed residue accumulation; and (3) multi-site, multi-year field validation studies to assess whether long-term, low-dose exposure leads to progressive herbicide accumulation in oilseed rape seeds, thereby informing the development of more scientifically grounded pesticide use guidelines.

## Data Availability

The data are contained within the article and Supplementary Materials.

## Supplementary Materials

The following supporting information can be downloaded at: https://www.mdpi.com/article/10.3390/antiox15080988/s1, Figure S1: Transcriptome analysis of rapeseed in response to HRM stress; Figure S2: Metabolomics analysis of rapeseed in response to HRM stress; Figure S3: Lipid analysis of rapeseed in response to HRM stress; Figure S4: Lipidomic profiles of germinating rapeseed seeds under HPM treatment; Table S1: Summary of RNA quality control results for samples used in RNA-seq analysis; Table S2: Summary of sample short reads after clearing from SGS sequencing; Table S3: Up- and downregulated genes in rapeseed with residual HP accumulation compared with control at 12 h after germination; Table S4: Up- and downregulated genes in rapeseed with residual HP accumulation compared with control at 24 h after germination; Table S5: Up- and downregulated metabolites in rapeseed with residual HP accumulation compared with control at 12 h after germination; Table S6: Up- and downregulated metabolites in rapeseed with residual HP accumulation compared with control at 24 h after germination; Table S7: Up- and downregulated lipids in rapeseed with residual HP accumulation compared with control at 12 h after germination; Table S8: Up- and downregulated lipids in rapeseed with residual HP accumulation compared with control at 24 h after germination; Table S9: FPKM Values and metabolites contents used to construct heat maps in various figures; Table S10: FPKM of differentially expressed genes (DEGs) in rapeseed with residual HPM accumulation compared with control; Table S11: Content of differentially expressed metabolites (DEMs) in rapeseed with residual HP accumulation compared with control; Table S12: Content of differentially expressed lipids (DELs) in rapeseed with residual HP accumulation compared with control.

## Abbreviations

The following abbreviations are used in this manuscript:

DEG	Differentially expressed gene
DEM	Differentially expressed metabolite
DEL	Differentially expressed lipid
GL	Glycerolipids
GP	Glycerophospholipids
SP	Sphingolipids
PR	Prenol lipids
ST	Sterol lipids
FA	Fatty acyls
SL	Saccharolipids
HPM	Haloxyfop-P-methyl
SOD	Superoxide dismutase
POD	Peroxidase
GSSG	Oxidized glutathione
GSH	Reduced glutathione
AsA	Ascorbic acid
DHA	Dehydroascorbic acid
