# Supplementary material for: Integrated Multi-Omics Analysis Reveals the Mechanism of Haloxyfop-P-methyl Residue Suppressing Seed Germination in Oilseed Rape (Brassica napus L.)
Source: Antioxidants (Basel). 2026 Aug 10;15(8):988. doi: 10.3390/antiox15080988 (PMC13509539; doi:10.3390/antiox15080988)
Supplement: Supplementary file 1 [file antioxidants-15-00988-s001.zip › Supplemental Figures.pdf]

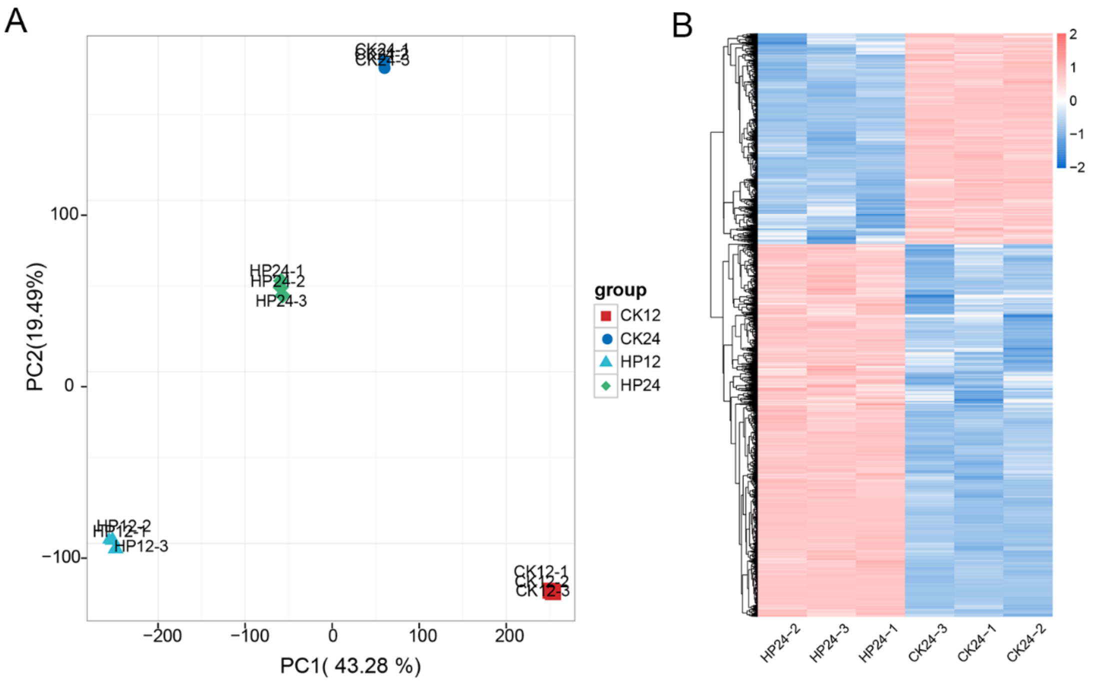


**Supplementary Figure S1. Transcriptome analysis of rapeseed in response to HRM stress**. (A) Principal component analysis of transcriptome data from control and HPM-treated rapeseed. (B) Heatmap of DEGs in rapeseed seeds under HPM stress at 12 h after germination. red: upregulated, blue: downregulated.

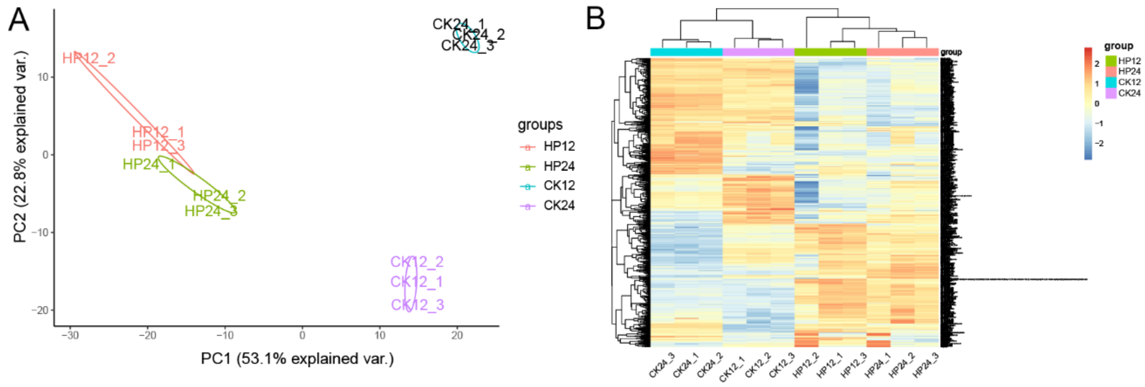


**Supplementary Figure S2. Metabolomics analysis of rapeseed in response to HRM stress**. (A) Principal component analysis of metabolomics data from control and HPM-treated rapeseed. (B) Heatmap of DEMs in rapeseed seeds under HPM stress.

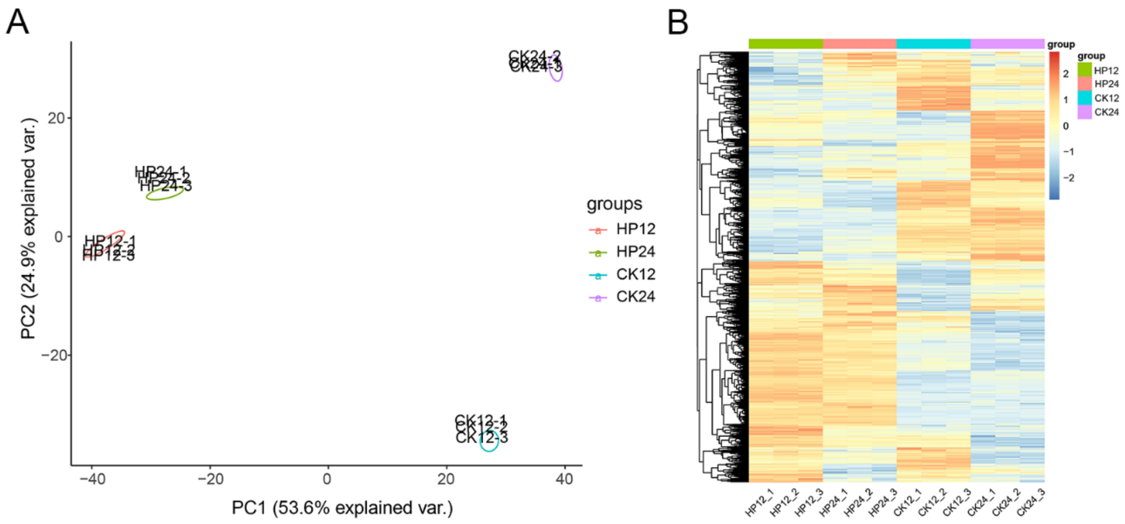


**Supplementary Figure S3. Lipids analysis of rapeseed in response to HRM stress**.

(A) Principal component analysis of lipids data from control and HPM-treated rapeseed.

(B) Heatmap of DELs in rapeseed seeds under HPM stress.

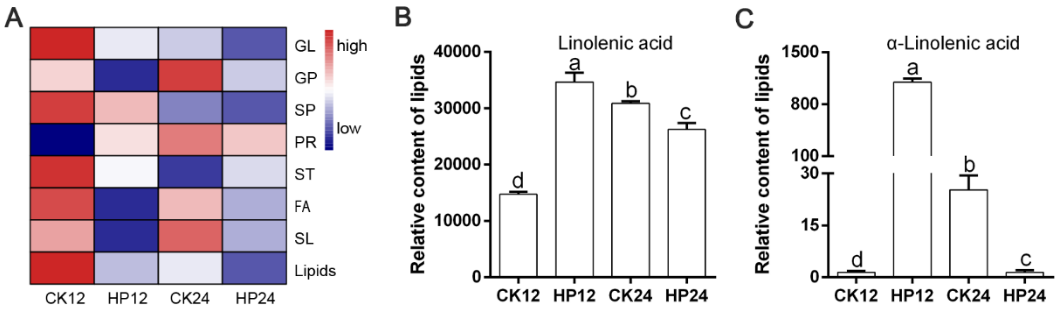


**Supplementary Figure S4. Lipidomic profiles of germinating rapeseed seeds under HPM treatment.** (A) Heatmap of significantly altered lipid species across eight major classes (FAs, GLs, GPs, SPs, STs, PRs, SLs, and PKs) in HPM-treated seeds compared with controls at 12 h and 24 h post-imbibition. Colors indicate relative abundance (red: increased; blue: decreased). (B) Relative abundance of linolenic acid in control and HPM-treated seeds at 12 h and 24 h. (C) Relative abundance of α-linolenic acid in control and HPM-treated seeds at 12 h and 24 h. Data are shown as mean ± SD (n = 3). Statistical significance was determined by one-way ANOVA combined with Tukey‘s test. Different letters indicate significant differences between groups ($p < 0.05$).
