# Supplementary material for: Integrated Multi-Omics Analysis Reveals the Mechanism of Haloxyfop-P-methyl Residue Suppressing Seed Germination in Oilseed Rape (Brassica napus L.)
Source: Antioxidants (Basel). 2026 Aug 10;15(8):988. doi: 10.3390/antiox15080988 (PMC13509539; doi:10.3390/antiox15080988)
Supplement: Supplementary file 1 [file antioxidants-15-00988-s001.zip › Supplemental Tables.pdf]

Table S1. Summary of RNA quality control results for samples used in RNA-seq analysis

Sample Name	Concentration(ng/ul)	OD260/280	OD260/230	RIN
CK12-1	715.9	2.17	1.43	8.7
CK12-2	713.9	2.18	1.43	8.7
CK12-3	715.4	2.17	1.42	8.7
CK24-1	909.2	2.18	1.86	8.4
CK24-2	908.1	2.16	1.86	8.6
CK24-3	907.6	2.16	1.89	8.6
HP12-1	235.8	2.21	0.65	8.2
HP12-2	234.5	2.12	0.62	8.1
HP12-3	236.45	2.21	0.66	8.1
HP24-1	296.6	2.25	0.64	8.6
HP24-2	297.5	2.23	0.63	8.5
HP24-3	295.6	2.24	0.62	8.6

Table S2. Summary of sample short reads of after clearing from SGS sequencing

Sample Name	Total Reads	Mapped Reads	Q20(%)	Q30(%)
CK12-1	45,191,340	41,943,600 (92.81%)	98.17	94.66
CK12-2	47,516,956	43,986,872 (92.57%)	98.14	94.69
CK12-3	40,769,356	37,878,967 (92.91%)	97.97	94.28
CK24-1	53,946,508	49,415,335 (91.60%)	97.89	94.04
CK24-2	50,013,220	46,173,022 (92.32%)	97.88	94.01
CK24-3	50,856,048	47,199,346 (92.81%)	98.16	94.7
HP12-1	49,301,742	42,708,609 (86.63%)	97.71	93.73
HP12-2	49,473,356	43,289,056 (87.50%)	97.97	94.33
HP12-3	49,875,186	43,848,854 (87.92%)	97.77	93.85
HP24-1	54,334,908	49,747,868 (91.56%)	97.87	93.9
HP24-2	50,971,728	46,632,409 (91.49%)	97.85	93.92
HP24-3	52,651,146	47,508,457 (90.23%)	98.06	94.44

Table S3-attached separately

Table S3. Up- and downregulated genes in rapeseed with residual HP accumulation compared with control at 12 hours after germination

Table S4-attached separately

Table S4. Up- and downregulated genes in rapeseed with residual HP accumulation compared with control at 24 hours after germination

Table S5-attached separately

Table S5. Up- and downregulated metabolites in rapeseed with residual HP accumulation compared with control at 12 hours after germination

Table S6-attached separately

Table S6. Up- and downregulated metabolites in rapeseed with residual HP accumulation compared with control at 24 hours after germination

Table S7-attached separately

Table S7. Up- and downregulated lipids in rapeseed with residual HP accumulation compared with control at 12 hours after germination

Table S8-attached separately

Table S8. Up- and downregulated lipids in rapeseed with residual HP accumulation compared with control at 24 hours after germination

Table S9-attached separately

Table S9. FPKM Values and metabolites contents used to construct heat maps in various figures.

Table S10 FPKM of Differentially expressed genes (DEGs) in rapeseed with residual HPM accumulation compared with control

Group	Gene ID	CK12	HP12	CK24	HP24
KAS1	BnaA02G0298900ZS	12.17	1.12	4.90	5.05
KAS1	BnaA06G0425000ZS	10.82	0.85	3.72	2.37
KAS1	BnaC02G0407200ZS	6.10	0.73	5.75	0.40
KAS1	BnaC07G0248000ZS	19.21	3.79	10.32	6.67
FAD3	BnaA04G0191900ZS	67.92	5.59	98.56	41.94
FAD3	BnaA05G0136600ZS	79.55	8.44	104.95	37.42
FAD3	BnaC04G0180200ZS	37.55	2.35	42.74	17.74
FAD3	BnaC04G0496200ZS	19.43	1.07	23.77	8.75
GAPDH	BnaC05G0392100ZS	5.06	1.21	0.29	0.69
mMDH1	BnaA05G0162400ZS	100.55	47.82	16.49	51.08
mMDH1	BnaA06G0013200ZS	147.13	76.44	36.59	95.82
mMDH1	BnaA08G0015400ZS	203.48	86.45	24.133	84.28
mMDH1	BnaC03G0797800ZS	103.84	32.73	13.97	39.64
mMDH1	BnaC06G0127500ZS	76.31	47.89	12.58	42.19
HKL1	BnaA07G0284900ZS	2.62	2.73	19.19	6.69
HKL1	BnaA02G0159200ZS	3.56	5.33	34.24	15.16
HKL1	BnaC02G0203300ZS	1.512	1.17	8.03	2.79
HKL1	BnaC06G0326800ZS	3.18	6.48	23.49	9.32
HKL2	BnaC05G0392100ZS	1.41	0.731	0.49	0.42

Table S11 Content of Differentially expressed metabolites (DEMs) in rapeseed with residual HP accumulation compared with control

Compound	CK12	HP12	CK24	HP24
HpOTrE	122.71	428.57	429.57	326.82
FA 18:3	886.21	193.41	1061.70	417.97
FA 18:3	14717.46	34654.05	30884.67	26210.36
norlinolenic acid	4.57	31.69	1093.49	1.32
LPA 18:2	662123.98	719743.64	1106614.78	618735.29
LPI 18:1	1115935.36	1225036.48	1826280.46	1115059.13
TriHOME	177314.49	321638.60	1211898.85	161357.63
diHODE	12.77	70.33	197.09	19.65
FA 18:2;O2	148.88	83.09	96.64	48.66
TG 20:2_20:4_22:0	3129.86	1827.17	5065.30	1922.85

Table S12 Content of Differentially expressed lipids (DELs) in rapeseed with residual HP accumulation compared with control

Compound	CK12	HP12	CK24	HP24
diHODE	12.77	70.33	197.09	19.65
FA 18:2;O2	148.88	83.09	96.64	48.66
TG 20:2_20:4_22:0	3129.86	1827.17	5065.30	1922.85
PE 16:0_18:3	18668.17	7487.38	9336.69	9639.63
PG 14:0;O-14:0;O	1271.72	44.27	66.83	180.63
